# Two Decades of Light-Chain Amyloidosis: Real-World Trends in Diagnosis, Treatment, and Survival

**DOI:** 10.14740/jh2202

**Published:** 2026-06-20

**Authors:** Grace Gorecki, Oyepeju Abioye, Jivtesh Singh, Maahin Parvez, Santoshini Adivi, Prerna Mewawalla, Arjun Lakshman, Santhosh Sadashiv

**Affiliations:** a Division of Internal Medicine, Allegheny Health Network, Pittsburgh, PA 15212, USA; b Division of Hematology, Allegheny Health Network, Pittsburgh, PA 15212, USA

**Keywords:** AL amyloidosis, Daratumumab, CD38-targeted therapy, Treatment outcome, ANDROMEDA

## Abstract

**Background:**

Systemic light-chain (AL) amyloidosis is a life-threatening disorder with historically poor outcomes. Daratumumab is a newer treatment for AL amyloidosis, but real-world trends in diagnosis, treatment, and survival are not well characterized.

**Methods:**

We conducted a retrospective cohort study using the TriNetX Global Collaborative Network, identifying adults with AL amyloidosis from 2006 to 2025. To improve specificity, patients with concurrent multiple myeloma, and other common amyloidosis subtypes were excluded. Survival analyses were stratified by Boston University cardiac stages. Propensity score matching balanced demographics and comorbidities.

**Results:**

We included 43,878 patients. Survival was improved in 2016–2025 vs. 2006–2015 (hazards ratio (HR) = 0.75; 95% confidence interval (CI), 0.68–0.83). Stage II patients showed the most pronounced gains (1-year HR = 0.66, P = 0.020; 10-year HR = 0.77, P = 0.022; median overall survival (OS), 2,009 vs. 1,517 days). Stage IIIa/IIIb showed no decade-level improvement. In the daratumumab era (2021–2025 vs. 2016–2020), stage I demonstrated markedly superior 5-year survival (HR = 0.59; log-rank P = 0.001; median OS, 1,549 vs. 965 days), and stage IIIb showed 51% lower 5-year mortality (odds ratio (OR), 0.49; P = 0.043). Direct comparison of Dara-CyBorD vs. CyBorD (n = 59 matched pairs) confirmed superior survival (3-year HR = 0.33; log-rank P < 0.0001; median OS not reached vs. 694 days). Sensitivity analyses using unstaged patients and AL-specific International Classification of Diseases, 10th Revision (ICD-10) codes yielded consistent results.

**Conclusions:**

Real-world outcomes in AL amyloidosis improved across two decades, with declining mortality and therapeutic shifts following daratumumab adoption. Most improvements occurred post-2021, underscoring CD38-targeted therapy’s transformative impact.

## Introduction

Systemic light-chain (AL) amyloidosis is a rare clonal plasma cell disorder that can cause considerable morbidity and mortality due to its rapidly progressive organ damage driven by amyloid fibril deposition [1]. Although its incidence has remained stable in US population studies—consistently estimated at 10–17 cases per million person-years—its prevalence has increased markedly, rising from 20.1 per million in 2007 to 50.1 per million in 2015 and reaching 69 per million in 2021 [2, 3]. This growing prevalence, despite stable incidence, reflects advances in disease recognition and improved survival in the modern therapeutic era, particularly following the introduction of daratumumab-based regimens in 2021.

Among affected organs, cardiac involvement is the dominant prognostic determinant, shaping treatment eligibility, response, and overall survival (OS) [4, 5]. Patients with AL amyloidosis rarely present with a suspected diagnosis; instead, they present with nonspecific symptoms, such as fatigue and dyspnea; organ-specific manifestations, including nephrotic-range proteinuria with edema (renal involvement, 60–70%), heart failure with preserved ejection fraction (cardiac involvement, 70–80%); or less commonly macroglossia, peripheral neuropathy, or hepatomegaly [1, 6]. Cardiac involvement is the leading cause of death, which explains why staging systems prioritize cardiac biomarkers despite renal symptoms often prompting initial evaluation [1].

Given this central prognostic role, staging systems have become critical tools for therapeutic decision-making. Staging and risk stratification are commonly performed using biomarkers of plasma cell dyscrasia and cardiac and kidney involvement. Commonly used staging and risk stratification models include the Mayo 2012 system, the Boston University staging system, and renal staging criteria [7–9]. The Boston University staging system was selected for our analysis due to its use of brain natriuretic peptide (BNP, rather than N-terminal pro–B-type natriuretic peptide (NT-proBNP)), which has broader availability in real-world practice and demonstrates high concordance with Mayo 2004 staging (κ = 0.854) [10].

Therapeutic innovation has further reshaped the AL amyloidosis landscape. Historically, the prognosis for AL amyloidosis was poor, particularly for patients with advanced cardiac involvement, and early mortality rates remained high despite bortezomib-based regimens [1, 11, 12]. AL amyloidosis arises from an abnormal clonal population of CD38-expressing plasma cells producing misfolded immunoglobulin light chains that circulate, deposit in tissues, and trigger progressive organ injury [13]. CD38-directed therapy offers a mechanism-based approach for more effective and sustained clonal suppression. The phase III ANDROMEDA trial established daratumumab-based therapy as a new standard by demonstrating a hematologic complete response rate of 53% versus 18% with the combination of cyclophosphamide, bortezomib, and dexamethasone (CyBorD) alone and a significant reduction in major organ deterioration, hematologic progression, or death [13]. This led to US Food and Drug Administration (FDA) approval of daratumumab-CyBorD (Dara-CyBorD) in January 2021, making it the only FDA-approved regimen for newly diagnosed AL amyloidosis and establishing it as a category 1, preferred, first-line therapy [14]. Real-world data have reinforced these results, reporting hematologic response rates exceeding 90%, rapid organ improvement, and substantial gains in event-free and OS [15, 16].

Despite these therapeutic advances, important knowledge gaps remain. Real-world trends in diagnosis stage, treatment adoption, and survival across decades have not been fully characterized following the introduction of daratumumab. In this study, we use a large dataset to examine temporal trends in diagnosis, staging, treatment patterns, and survival in systemic AL amyloidosis.

## Materials and Methods

This retrospective cohort study utilized the TriNetX Global Collaborative Network analytics platform, a federated network that aggregates deidentified electronic health records and is therefore exempt from the Institutional Review Board (IRB) approval. This study was conducted in compliance with the ethical standards of the responsible institution on human subjects, as well as with the Helsinki Declaration. The TriNetX platform complies with the Health Insurance Portability and Accountability Act (HIPAA) and has been granted a waiver of informed consent by the Western IRB due to the aggregated nature of the data and provision of only statistical summaries of deidentified information. Patients diagnosed with systemic amyloidosis were identified using International Classification of Diseases, 10th Revision (ICD-10) code E85 between January 1, 2006, and December 31, 2025. To improve specificity for AL amyloidosis, we applied the following exclusion criteria: concurrent diagnosis of multiple myeloma (ICD-10 code C90.0); and diagnosis codes for non-AL amyloidosis subtypes, including hereditary amyloidosis (E85.2), organ-limited amyloidosis (E85.4), and transthyretin-related amyloidosis (E85.82).

To evaluate temporal trends, patients were stratified into three comparison groups: (1) a primary decade comparison (2006–2015 vs. 2016–2025) to assess long-term changes in diagnosis and outcomes; (2) a daratumumab era comparison (2016–2020 vs. 2021–2025) to evaluate outcomes following FDA approval in January 2021; and (3) a direct treatment comparison of Dara-CyBorD versus CyBorD alone as frontline therapy. Also, two sensitivity analyses were performed: (1) an analysis restricted to unstaged patients to assess whether survival trends were consistent independent of staging ascertainment; and (2) an analysis restricted to patients coded specifically with AL amyloidosis (ICD-10 E85.81) to evaluate the robustness of findings to case definition.

Cardiac staging was performed using the Boston University staging system. Staging was derived from laboratory values documented in electronic health records using the following thresholds: stage I (BNP ≤ 81 pg/mL and troponin I ≤ 0.1 ng/mL), stage II (BNP > 81 pg/mL or troponin I > 0.1 ng/mL, but not meeting stage III criteria), stage IIIa (BNP > 81 pg/mL and troponin I > 0.1 ng/mL with BNP ≤ 700 pg/mL), and stage IIIb (BNP > 700 pg/mL and troponin I > 0.1 ng/mL).

The primary outcome was all-cause mortality, with secondary outcomes including landmark mortality at 1, 3, 5, and 10 years, and median OS. To minimize confounding, 1:1 propensity score matching was performed using age, sex, race, and comorbidities (diabetes mellitus, hypertension, chronic kidney disease, heart failure, ischemic heart disease, and malignancy), with covariate balance confirmed using standardized mean differences (< 0.1 indicating adequate balance). Survival was estimated using Kaplan–Meier analysis with log-rank testing. Results are reported as hazard ratios (HRs) and odds ratios (ORs) with 95% confidence intervals (CIs). HRs were derived from Cox regression and ORs from logistic regression. All tests were two-sided, with P < 0.05 considered statistically significant. Analyses were conducted using the TriNetX Advanced Analytics Platform.

## Results

**Figure 1:**
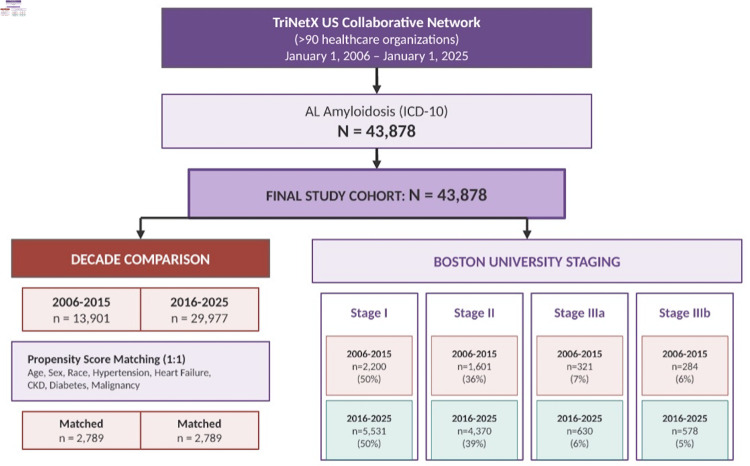
Flow diagram of patient selection, analysis cohorts, and staging distribution. Patients with systemic amyloidosis were identified from the TriNetX US Collaborative Network using International Classification of Diseases, 10th Revision (ICD-10) code E85 between January 1, 2006, and December 31, 2025. Propensity score matching (1:1) was performed using age, sex, race, and comorbidities, yielding 2,789 matched patients per group.

A total of 43,878 patients with AL amyloidosis meeting inclusion criteria were identified from 2006 to 2025. The 2006–2015 cohort included 13,901 patients and the 2016–2025 cohort included 29,977 patients, representing a 2.2-fold increase in diagnosed cases (Fig. 1). Cardiac staging data were available for 4,406 of 13,901 patients (31.7%) in the 2006–2015 cohort and 11,109 of 29,977 patients (37.1%) in the 2016–2025 cohort (Table 1). Among staged patients, stage I was most common (2,200 vs. 5,531), followed by stage II (1,601 vs. 4,370), stage IIIa (321 vs. 630), and stage IIIb (284 vs. 578). The proportion of stage II cases increased from 11.5% to 14.6% across decades.

**Table 1:** Baseline Characteristics of Patients Diagnosed With Systemic AL Amyloidosis in 2006–2015 and 2016–2025 Before and After Propensity Score Matching^a^

	2016–2025 cohort (n = 27,198)	2006–2015 cohort (n = 12,659)	P value	SMD
Age at index (years), mean ± SD	61.1 ± 20.7	56.6 ± 23.5	0.001	0.202
Current age (years), mean ± SD	65.4 ± 20.0	69.3 ± 22.6	0.001	0.183
Age at index (years), median (IQR)	66 (24)	63 (28)	–	–
Race, n (%)				
White	14,320 (52.7)	7,259 (58.0)	0.001	0.108
Black or African American	3,753 (13.8)	1,258 (10.0)	0.001	0.116
Asian	989 (3.6)	1,107 (8.8)	0.001	0.217
American Indian or Alaska Native	91 (0.3)	36 (0.3)	0.441	0.008
Native Hawaiian or Other Pacific Islander	85 (0.3)	26 (0.2)	0.066	0.021
Other	797 (2.9)	392 (3.1)	0.275	0.012
Unknown	7,163 (26.3)	2,440 (19.5)	0.001	0.163
Ethnicity, n (%)				
Not Hispanic or Latino	17,301 (63.6)	8,224 (65.7)	0.001	0.044
Unknown	8,776 (32.3)	3,609 (28.8)	0.001	0.075
Comorbidities, n (%)				
Heart failure	3,744 (13.8)	1,276 (10.2)	0.001	0.11
Chronic kidney disease	3,445 (12.7)	1,166 (9.3)	0.001	0.107
Hypertensive diseases	6,284 (23.1)	3,116 (24.9)	0.001	0.042
Diabetes mellitus	3,362 (12.4)	1,057 (8.4)	0.001	0.129
Neoplasms	3,152 (11.6)	1,285 (10.3)	0.001	0.042
Atherosclerotic heart disease	1,756 (6.5)	785 (6.3)	0.483	0.008

^a^Propensity score matching (1:1) was performed using age, sex, race, and major comorbidities (diabetes mellitus, hypertension, chronic kidney disease, heart failure, ischemic heart disease, and malignancy). Covariate balance was assessed using SMD, with values < 0.1 indicating adequate balance. Cardiac stage distribution is according to the Boston University staging system. SD: standard deviation; IQR: interquartile range; SMD: standardized mean differences.

**Figure 2:**
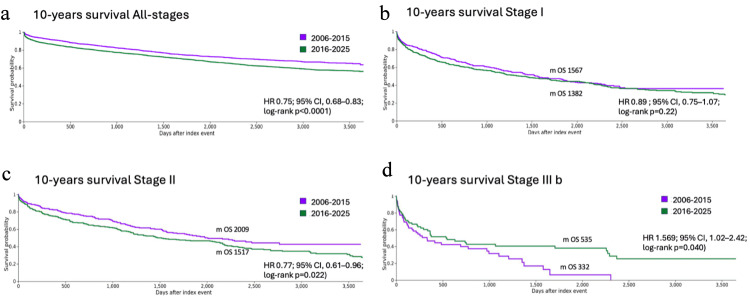
Kaplan–Meier overall survival (OS) analyses based on decade (2006–2015 vs. 2016–2025) and disease state after propensity score matching. (a) All stages. (b) Stage I. (c) Stage II. (d) Stage IIIb. HR: hazard ratio; CI: confidence interval.

When comparing the two decades, all-cause mortality declined significantly in the modern decade across all timepoints. Kaplan–Meier analysis confirmed significantly superior survival in the modern decade (HR = 0.75; 95% CI, 0.68–0.83; log-rank P < 0.0001) (Fig. 2a), with median OS not reached in either cohort. All-cause mortality was consistently lower across all landmark timepoints, with 33–34% relative reductions at both 1-year (OR = 0.67; 95% CI, 0.57–0.79; P < 0.0001) and 10-year (OR = 0.66; 95% CI, 0.58–0.74; P < 0.0001).

Among stage I patients, survival trended toward improvement in the modern decade (HR = 0.89; 95% CI, 0.68–1.07; log-rank P = 0.22), with median OS improving from 1,382 to 1,567 days, a 185-day gain (Fig. 2b). One-year mortality showed a borderline significant reduction (OR = 0.75; 95% CI, 0.57–1.00; P = 0.049), while 10-year mortality was markedly improved (OR = 0.67; 95% CI, 0.53–0.86; P = 0.002). Patients with stage II cardiac disease demonstrated the most consistent improvements. Survival was significantly superior in the modern decade at all timepoints: 1-year (HR = 0.66; log-rank P = 0.020), 5-year (HR = 0.78; log-rank P = 0.039), and 10-year (HR = 0.77; 95% CI, 0.61–0.96; P = 0.022), with median OS improving from 1,517 to 2,009, a 492-day (16-month) gain (Fig. 2c). Ten-year mortality showed the most pronounced benefit (OR = 0.55; 95% CI, 0.40–0.74; P < 0.0001). Among stage IIIa patients, no significant differences in mortality were observed at any timepoint. Although the OR for stage IIIb mortality did not reach significance (OR = 1.23; P = 0.562), Kaplan–Meier analysis revealed a trend toward worse survival in the modern decade (median OS 332 vs. 535 days; HR = 1.57; 95% CI, 1.02–2.42; log-rank P = 0.04) (Fig. 2d).

**Figure 3:**
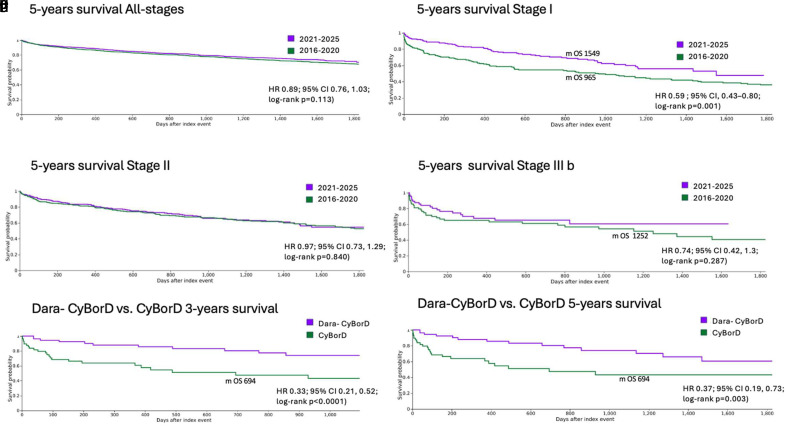
Kaplan–Meier analysis curves comparing overall survival (OS) before (2016–2020) and after (2021–2025) the introduction of daratumumab-based therapy. (a) All stages. (b) Stage I. (c) Stage II. (d) Stage IIIb. (e, f) Treatment comparison. Patients receiving Dara-CyBorD demonstrated improved 3-year, and 5-year OS compared with CyBorD alone. HR: hazard ratio; CI: confidence interval.

To evaluate outcomes following FDA approval of daratumumab (January 2021), we compared patients diagnosed in 2016–2020 with those diagnosed in 2021–2025. Kaplan–Meier analysis showed a trend toward improved survival (HR = 0.89; log-rank P = 0.113) (Fig. 3a). Mortality reductions emerged progressively over time, with a borderline reduction at 1 year (OR = 0.80; 95% CI, 0.64–1.00; P = 0.054), and a 26% relative reduction at 5 years (OR = 0.74; 95% CI, 0.63–0.88; P < 0.0005).

Stage I patients showed particularly striking improvements in the daratumumab era. Survival was markedly superior at both 1 year (HR = 0.42; log-rank P = 0.0001) and 5 years (HR = 0.59; 95% CI, 0.43–0.80; log-rank P = 0.001), with median OS improving from 965 to 1,549 days, a 584-day (19-month) gain (Fig. 3b). Corresponding ORs confirmed a 62% relative reduction in 1-year mortality (OR = 0.38; 95% CI, 0.24–0.61; P < 0.0001) and 57% at 5 years (OR = 0.43; 95% CI, 0.29–0.64; P < 0.0001). For stage II patients, 5-year mortality showed a trend toward improvement (OR = 0.71; 95% CI, 0.51–1.01; P = 0.053), but Kaplan–Meier analysis showed no significant difference (HR = 0.97; 95% CI, 0.73–1.29; log-rank P = 0.840) (Fig. 3c). No significant differences were observed for stage IIIa patients at any timepoint. Among stage IIIb patients, 5-year mortality was significantly lower in the daratumumab era (OR = 0.49; 95% CI, 0.25–0.98; P = 0.043), representing a 51% relative reduction. Median OS was not reached vs. 1,252 days (41 months), though Kaplan–Meier analysis did not reach significance (HR = 0.74; 95% CI, 0.42–1.30; log-rank P = 0.287) (Fig. 3d).

**Figure 4:**
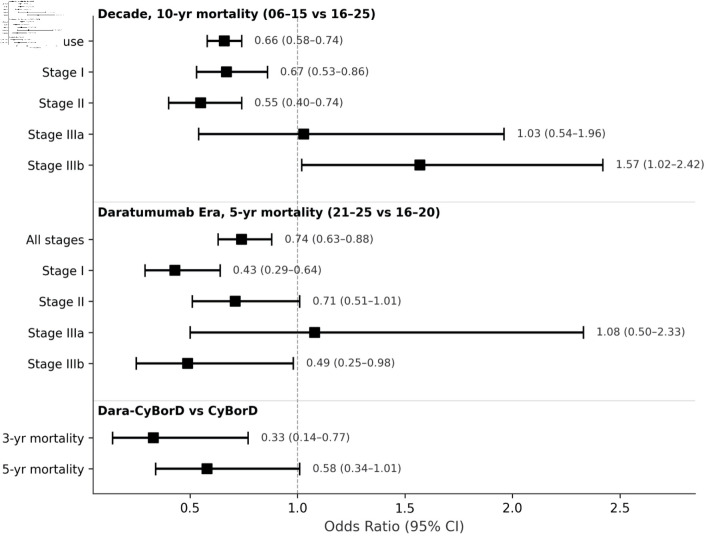
Forest plot of mortality outcomes by era and disease stage. Odds ratios (OR) with 95% CIs for mortality comparing patients diagnosed in 2006–2015 vs. 2016–2025 (10-year mortality) and patients treated in the pre-daratumumab (2016–2020) vs. daratumumab (2021–2025) eras (5-year mortality), stratified by Boston University cardiac stage. Additional analyses compare outcomes with Dara-CyBorD vs. CyBorD for 3-year and 5-year mortality. The dashed vertical line indicates OR = 1.0 (no difference). Values < 1 favor the modern or daratumumab era cohort. CI: confidence interval; yr: year.

To directly evaluate the impact of daratumumab addition to standard therapy, we compared patients who received Dara-CyBorD versus CyBorD alone as frontline therapy. Kaplan–Meier analysis confirmed markedly superior survival with Dara-CyBorD at both 3 years (HR = 0.33; 95% CI, 0.21–0.52; log-rank P < 0.0001) (Fig. 3e) and 5 years (HR = 0.37; 95% CI, 0.19–0.73; log-rank P = 0.003) (Fig. 3f). Three-year mortality was 67% lower with Dara-CyBorD (OR = 0.33; 95% CI, 0.14–0.77; P = 0.009), and 5-year mortality was 42% lower (OR = 0.58; 95% CI, 0.34–1.01; P = 0.048). Median OS was 694 days (23 months) in the CyBorD cohort versus not reached in the Dara-CyBorD cohort. A forest plot summarizing mortality outcome (OR) across all study comparisons is presented in Figure 4.

We also performed two sensitivity analyses to support our findings. First, among unstaged patients (those without available cardiac biomarker data for Boston University staging), survival trends mirrored the primary analysis. In the decade comparison, 10-year survival was significantly improved in the modern decade (HR = 0.79; 95% CI, 0.68–0.92; log-rank P = 0.003). Survival was significantly superior in the post-2021 cohort (HR = 0.77; 95% CI, 0.62–0.95; log-rank P = 0.014). Second, we restricted the analysis to patients identified using the AL-specific ICD-10 code. In the post-2021 era comparison, survival was significantly improved in the most recent era (HR = 0.78; 95% CI, 0.63–0.95), consistent with the primary analysis. The decade comparison using the AL-specific code was limited by a substantial cohort imbalance (n = 614 in 2006–2015 vs. n = 11,691 in 2016–2025).

## Discussion

In our study, we observed substantial improvements in survival across the modern treatment era. The number of diagnosed cases increased 2.2-fold, consistent with improved disease recognition, enhanced screening, and the widespread availability of advanced diagnostic modalities [1, 17, 18]. All-cause mortality declined significantly across all timepoints, with 33–34% relative reductions at both 1-year and 10-year landmarks (both P < 0.0001). Kaplan–Meier analysis confirmed superior survival in the modern decade (HR = 0.75; log-rank P < 0.0001). These improvements coincide temporally with the introduction of daratumumab-based regimens. In addition, mass-spectrometry–based amyloid typing and noninvasive imaging techniques, including cardiac magnetic resonance imaging (MRI) and bone-avid tracer scintigraphy, have significantly increased diagnostic accuracy and enabled earlier identification of systemic involvement [13, 19]. Early diagnosis remains one of the strongest prognostic determinants in AL amyloidosis, as earlier intervention may prevent irreversible cardiac injury [1, 13, 14, 16, 20].

Survival gains were particularly pronounced in stage II patients, who demonstrated the most consistent improvements across all timepoints, with significant mortality and median OS improving by 492 days (16 months). This finding aligns with a recent Mayo Clinic study of 361 patients comparing Dara-CyBorD versus CyBorD, which similarly found the most pronounced survival benefit in stage II disease, with significantly higher 2-year OS: 97.6% vs. 78.9% (HR = 0.10; 95% CI, 0.04–0.24; P = 0.004) [21]. The intermediate cardiac involvement in stage II may represent an optimal therapeutic window, with sufficient organ reserve to tolerate treatment while still having reversible dysfunction amenable to therapy.

Stage I patients showed particularly striking improvements in our post 2021 analysis, with median OS improving by 584 days. These findings are consistent with the ANDROMEDA trial, which demonstrated hematologic complete response rates of 53.3% with Dara-CyBorD versus 18.1% with CyBorD alone, along with significantly higher cardiac (41.5% vs. 22.2%) and renal (53.0% vs. 23.9%) response rates at 6 months [13].

Stage IIIb patients—a population excluded from the ANDROMEDA trial—showed a 51% reduction in 5-year mortality in the post-2021 daratumumab era analysis (OR = 0.49; P = 0.043), with median OS not reached versus 1,252 days. This finding is clinically meaningful given that stage IIIb disease historically carries the poorest prognosis, with median survival often measured in months. In our decade comparison, Kaplan–Meier analysis revealed paradoxically worse survival in the modern decade (median OS, 332 vs. 535 days). This discordance likely reflects stage migration, as enhanced diagnostic sensitivity identifies more patients with advanced, rapidly progressive disease who previously died undiagnosed. While treatment-related toxicity in this fragile population cannot be entirely excluded, a deleterious treatment effect is unlikely, given that the same population showed significant improvement in the daratumumab era, consistent with external data demonstrating superior survival with daratumumab-based therapy in stage IIIb disease [22].

Our direct comparison of Dara-CyBorD versus CyBorD further supports that the survival improvements observed after 2021 are at least partly attributable to daratumumab introduction, with median OS not reached in the Dara-CyBorD cohort versus 694 days with CyBorD alone. However, given the small, matched sample size and inability to adjust for key clinical variables such as cardiac stage and frailty, these results should be considered hypothesis-generating. Nevertheless, the large effect size and consistency with external data from the ANDROMEDA trial and the Mayo Clinic retrospective study support the biological plausibility of these findings.

These real-world findings are corroborated by multiple external studies. A single-center experience of 99 patients demonstrated that daratumumab plus proteasome inhibitor–based regimens achieved higher complete response rates compared with proteasome inhibitor–based and chemotherapy regimens, respectively, with rapid time to response (median 1 month to partial response or better) [15]. Similarly, the Mayo Clinic study of 361 patients demonstrated higher 2-month hematologic response rates (60.8% vs. 31.1%; P < 0.001), lower 6-month mortality (8.8% vs. 16.3%; P = 0.04), and superior OS with Dara-CyBorD [21]. Additional observational data have confirmed daratumumab efficacy in both newly diagnosed and relapsed/refractory settings, with organ responses observed even in previously treated patients [23, 24]. Together, these findings reinforce the transformative impact of daratumumab addition to standard therapy and support its current role as the preferred frontline regimen.

Despite these advances, important gaps, particularly the persistent disconnect between hematologic response and organ recovery, remain a challenge limiting further survival gains. Real-world studies consistently show that even when deep hematologic responses are achieved, organ recovery—especially cardiac—lags significantly behind; in one study, only 46% of newly diagnosed patients treated with daratumumab demonstrated cardiac response at 6 months [22].

These findings underscore the need for next-generation strategies that complement clonal suppression with accelerated fibril clearance. Monoclonal antibodies and small molecules targeting amyloid deposits directly represent promising candidates currently under investigation [1, 25]. Additionally, plasma cell–directed immunotherapies such as chimeric antigen receptor (CAR)-T cells and bispecific antibodies may achieve deeper and more durable hematologic responses in selected patients, particularly those with relapsed or refractory disease [26].

Finally, given that amyloid deposition accumulates over years before clinical presentation, early detection remains critical to maximizing therapeutic benefit. Emerging artificial intelligence tools show substantial promise in this domain: artificial intelligence-enhanced echocardiography models have achieved an area under the curve of 0.93 for detecting cardiac amyloidosis, outperforming traditional clinical algorithms and improving referral of high-risk patients while reducing unnecessary downstream testing [27]. Integration of such approaches into routine practice may meaningfully shift diagnosis to earlier, more treatable disease stages.

### Limitations

Several limitations warrant consideration. As a retrospective analysis using real-world data, observed trends may have been influenced by unmeasured confounding related to institutional practices, referral patterns, and differences in diagnostic availability. Despite applying exclusion criteria to enrich for AL amyloidosis, some misclassification may have occurred, as bone marrow plasma cell percentage and bone lesions could not be reliably captured in the TriNetX database. Organ-specific response data were unavailable, limiting our ability to correlate survival improvements with cardiac or renal recovery. The Dara-CyBorD versus CyBorD comparison included a relatively small, matched cohort (n = 59 per group), which may limit statistical power to detect differences at certain timepoints despite the striking survival separation observed. Staging data were unavailable for approximately 30% of patients, reflecting real-world variability in biomarker documentation; however, staged and unstaged patients showed similar baseline characteristics. Numbers at risk at specific Kaplan–Meier timepoints were not available through the TriNetX platform, though the large cohort sizes and consistency of findings across multiple landmarks support the validity of our survival analyses. Key disease-specific parameters including differential free light-chain (dFLC) levels, bone marrow plasma cell percentage, detailed organ involvement mapping, and hematologic and organ response assessments are not captured in the TriNetX database. This trade-off is inherent to large administrative databases, which prioritize sample size and generalizability over granular clinical detail.

### Conclusions

Our study demonstrates substantial real-world improvement in systemic AL amyloidosis outcomes over the past two decades, characterized by earlier diagnosis, increased use of daratumumab-based regimens, and marked reductions in mortality, particularly in high-risk patients. Despite these advances, persistent gaps remain between hematologic response and organ recovery. Future progress will require integration of earlier detection strategies, personalized risk-adapted therapy, and development of fibril-targeted or immune-based treatments to further optimize outcomes and extend survival in this complex disease.

## Data Availability

The data supporting the findings of this study are available from the corresponding author upon reasonable request.

## Abbreviations

AL: light-chain
BNP: brain natriuretic peptide
CI: confidence interval
FDA: Food and Drug Administration
HIPAA: Health Insurance Portability and Accountability Act
HR: hazard ratio
ICD-10: International Classification of Diseases, 10th Revision
MRI: magnetic resonance imaging
OR: odds ratio
OS: overall survival
SMD: standardized mean differences
